# Could digital patient communities be the launch pad for patient-centric trial design?

**DOI:** 10.1186/1745-6215-15-172

**Published:** 2014-05-15

**Authors:** Paul Wicks

**Affiliations:** 1 PatientsLikeMe, 155 2nd Street, Cambridge, MA 02141, USA

**Keywords:** Patient centered trial design, Patient powered research networks, Protocol design, Clinical trials

## Abstract

The system of medical discovery does not revolve around patients as unique individuals with preferences, needs, and desires. Rather it revolves around scientific scrutiny, the needs of the sponsor, and the desires for regulatory approval. The patient is only a subject. Is it any wonder, then, that some patients have rejected the current medical paradigm and sought to find their own path?

## Background

For an individual used to the uncanny intelligence that drives the personalized content you see on Amazon or Google, the experience of being a clinical trial participant must seem like a blast from the 1980s. Rather than receiving a welcoming email that alerts you to upcoming trials, you might get a letter in the post that has so much medical jargon it can only be understood by a scientific expert. Or you might have a hasty conversation with your doctor who knows few of the details and has even less time to explain them.

The system of medical discovery does not revolve around patients as unique individuals with preferences, needs, and desires. Rather it revolves around scientific scrutiny, the needs of the sponsor, and their desire for regulatory approval. The patient is only a subject. Is it any wonder, then, that some patients have rejected the current medical paradigm and sought to find their own path?

## Participant led research

In the rare and terminal condition amyotrophic lateral sclerosis (ALS) there has been evidence from as far back as 2004 [1] of patients violating protocol by taking experimental creatine supplements off-label when they were meant to be on the placebo arm. More recently, when a small phase II study reported that lithium carbonate might halt the progression of the disease in a small sample of 16 patients treated with lithium and riluzole relative to 28 patients on riluzole alone, patients rapidly mobilized to get hold of lithium off-label from their physicians. Within just six months a group of 160 patients had convened online and were tracking their disease progression, lithium blood levels, and side effects to discover whether they could crowdsource a study faster than the traditional medical establishment [2]. In the end, an analysis performed by PatientsLikeMe showed that lithium had no effect [3], a finding confirmed by a number of clinical trials in later years [4]. Some patients even went on to perform similar attempts at crowdsourcing while participating in randomized controlled trials, pooling their side effects to try and unblind themselves [5]. Scientifically these protocol violations are a concern, but from an advocate’s perspective they seem like a reasonable reaction. Might there be a middle ground?

### Patient centered trial design

A promising example from the iSPY-2 breast cancer trial suggests that integrating patients as informed decision makers rather than strictly as subjects may optimize study design, create better recruitment materials, minimize attrition, and ensure meaningful dissemination of trial results to women affected by the disease. Although it took more time and resources, the study investigators recruited up to 50 patients to advise, co-design, translate recruitment materials into other languages, and even sit on scientific advisory committees within the trial infrastructure [6]. As a result blind spots were illuminated by patients before recruitment started and researchers managed to successfully enroll participants in a study with a fairly invasive protocol of repeated biopsies and imaging.

Another example comes from the Outcome Measures in Rheumatology (OMERACT) group, which invited rheumatoid arthritis (RA) patient advocates to participate in their bi-annual meetings to identify important outcome measures. Outcomes that matter to patients that had previously flown under the radar, such as fatigue, flare-ups, sleep disturbance, and foot pain were identified. Furthermore, advocates reported a cultural change that arises from having patients in the room when decisions are being made [7].

## Discussion

As enticing as these examples sound, it is worth noting that they are the exception rather than the rule. This is in part due to the cultural and logistical issues involved [6,8]. One potential advantage of newer digital technology is that it might be possible to scale patient involvement in a way that is not overly burdensome for either patient or researcher, and that can entice ongoing involvement throughout the trial. For example, we have found that by iterating on digital feedback mechanisms, we can bring the time needed for certain aspects of psychometric validation for patient-reported outcome measures down from months to days [9], with online patients eager to offer feedback if tools are convenient and engaging.

Some early efforts are underway at the earliest end of the funnel, awareness. Charities and non-profits such as the Michael J Fox Foundation have built tools like the Fox Trial Finder (https://foxtrialfinder.michaeljfox.org/), which explains the importance of trials, and hosts lay summaries of many clinical trials, albeit mostly in the United States. Online startup companies such as TrialReach.com attempt to match patients to trials and provide services to help provide lay summaries and reach out to patients where they are. PatientsLikeMe pulls in all the data from ClinicalTrials.gov each night and matches it to over 250,000 globally registered patients in their system so that those who want to can find local trials that match their clinical profile. The trial registries themselves are making strides to improve the ability of patients and researchers to come together, and the ISRCTN Register (hosted by Biomed Central) are exploring new ways to support researchers in providing lay summaries and research feedback.

## Conclusion

By harnessing online patient communities it is possible to recruit a representative population of dozens or even hundreds of patients to provide qualitative and quantitative feedback at each phase of the trial recruitment process. Direct patient input and involvement about the relative importance of research questions, the unmet need in their disease, potential barriers to recruitment in trial protocols recruitment materials, and, once completed, even the writing of the lay summary of trial results can improve public understanding of science. Such tools might only be applicable to certain diseases with high enough levels of online engagement, but the status quo is that for very few conditions do trials have any sort of patient involvement or co-design. Given the well-documented struggles many trials have to meet their targets, it at least warrants experimentation. The advantage in doing so with software versus traditional models is a more rapid iteration in response to feedback and learning, though there are likely to be downsides to over-reliance on purely digital methods, such as biases, which will need to be addressed [10].

If patients are increasingly comfortable sharing their data and providing feedback as consumers in areas as diverse as shopping, hotels, and restaurants, what have we got to lose in trying to engage them as peers in designing better research?

## Abbreviations

ALS: amyotrophic lateral sclerosis; iSPY-2: Investigation of Serial Studies to Predict Your Therapeutic Response with Imaging And Molecular Analysis 2); OMERACT: Outcome Measures in Rheumatology group; RA: rheumatoid arthritis.

## Competing interests

The author has no competing interests. PW is an employee of PatientsLikeMe and holds stock/stock options in the company. The PatientsLikeMe research team has received research support from Accorda, Abbott, the AKU Society, Avanir, Biogen, Boehringer Ingelheim, Genzyme, Janssen, Johnson & Johnson, Kaiser Permanente, Merck, Novartis, Otsuka, the Robert Wood Johnson Foundation, Sanofi, University of Maryland, University of Michigan and UCB. PW sits on the Advisory Board of Current Controlled Trials, which administers the ISRCTN Register, and the Editorial Advisory Board of the BMJ.

## References

[B1] Shefner JM Cudkowicz ME Schoenfeld D Conrad T Taft J Chilton M Urbinelli L Qureshi M Zhang H Pestronk A Caress J Donofrio P Sorenson E Bradley W Lomen-Hoerth C Pioro E Rezania K Ross M Pascuzzi R Heiman-Patterson T Tandan R Mitsumoto H Rothstein J Smith-Palmer T MacDonald D Burke D NEALS Consortium: a clinical trial of creatine in ALS Neurology 2004 63 1656 1661 10.1212/01.WNL.0000142992.81995.F0 15534251

[B2] Frost JH Massagli MP Wicks P Heywood J How the social web supports patient experimentation with a new therapy: The demand for patient-controlled and patient-centered informatics AMIA Annu Symp Proc 2008 217 221 http://www.ncbi.nlm.nih.gov/pmc/articles/PMC2656086/18999176 PMC2656086

[B3] Wicks P Vaughan TE Massagli MP Heywood J Accelerated clinical discovery using self-reported patient data collected online and a patient-matching algorithm Nat Biotechnol 2011 29 411 414 10.1038/nbt.1837 21516084

[B4] Armon C Is the lithium-for-ALS genie back in the bottle? Not quite Neurology 2010 75 586 587 10.1212/WNL.0b013e3181ed9ef7 20702793

[B5] Wicks P Vaughan T Heywood J Subjects no more: what happens when trial participants realize they hold the power? BMJ: Br Med J 2014 348 g368 10.1136/bmj.g368 PMC3905107 24472779

[B6] Perlmutter J Advocate involvement in I-SPY 2 Breast Dis Year Bk Q 2011 22 21 24 10.1016/j.breastdis.2011.01.045

[B7] de Wit M Abma T Loon MK-V Collins S Kirwan J Involving patient research partners has a significant impact on outcomes research: a responsive evaluation of the international OMERACT conferences BMJ Open 2013 3 e002241 e002241 23667160 10.1136/bmjopen-2012-002241 PMC3651970

[B8] de Wit MPT Koelewijn-van Loon MS Collins S Abma TA Kirwan J “If i wasn’t this robust”: patients’ expectations and experiences at the Outcome Measures in Rheumatology Conference 2010 Patient 2013 6 179 187 10.1007/s40271-013-0017-0 23736943

[B9] Wicks P Heywood B Heywood J Online platform to accelerate patient involvement in open instrument development Quality Life Res, 1 2013 22 S1 55 doi:10.1007/s11136-013-0543-1

[B10] Bove R Secor E Vaughan T Wicks P Glanz B Weiner H Chitnis T De Jager P Comparison of demographic and disease characteristics in patients with multiple sclerosis at an MS clinic and on an online research forum Neurology 2012 78 P01.145

